# Global prevalence, characteristics, and future prospects of IncX3 plasmids: A review

**DOI:** 10.3389/fmicb.2022.979558

**Published:** 2022-09-06

**Authors:** Xiaobing Guo, Ruyan Chen, Qian Wang, Chenyu Li, Haoyu Ge, Jie Qiao, Yuan Li

**Affiliations:** ^1^Department of Laboratory Medicine, The First Affiliated Hospital of Zhengzhou University, Zhengzhou, China; ^2^Department of Laboratory Medicine, The First Affiliated Hospital of Henan University of Chinese Medicine, Zhengzhou, China; ^3^Department of Nuclear Medicine, The First Affiliated Hospital of Zhengzhou University, Zhengzhou, China

**Keywords:** IncX3, plasmid, epidemiology, carbapenemases, drug resistance

## Abstract

IncX3 plasmids are narrow host range plasmids mostly found in *Enterobacteriaceae* with great conjugation ability, high stability, no fitness cost, and the ability to improve biofilm formation in their bacterial hosts. IncX3 plasmids have spread swiftly, primarily in several nations and among different species over the last 10 years. *bla_NDM_*, *bla_KPC_*, and *bla_OXA-181_* are the carbapenemase genes carried by IncX3 plasmids. Among them, *bla_NDM_* is often located on the IncX3 plasmid, which is deemed as the primary vehicle of *bla_NDM_* transmission. Isolates harboring IncX3 plasmids are found in nations all over the world from human, animal, and environmental sources. Cointegrate plasmids related to IncX3 have recently been discovered to increase the antibiotic resistance spectrum and potentially broaden the host range of plasmids, restricting the use of antibiotics in the clinic. There are, however, few reviews based on the physiological and epidemiological properties of IncX3 plasmid, as well as studies on the plasmid itself. Hence, we conducted a retrospective literature review to summarize the characteristics of IncX3 plasmids aiming to provide a theoretical basis for controlling the global prevalence of IncX3 plasmids and directions for further research on the functions of the related genes on the IncX3 plasmid.

## Introduction

Carbapenem antibiotics are often the last line of defense in the treatment of multidrug-resistant Gram-negative bacteria. However, with the widespread use of antibiotics, carbapenem-resistant *Enterobacteriaceae* (CRE) have become common and pose a great threat to human health. Phenotypic resistance to carbapenems is complex and mainly caused by four mechanisms, including (1) β-lactamase activity combined with structural mutations; (2) production of carbapenemases, enzymes that hydrolyze carbapenem antibiotics; (3) activation and massive expression of drug efflux pump system; and (4) alterations in penicillin-binding proteins. The most typical mechanism is carbapenemase production (KPC, NDM, VIM, IMP, and OXA-48; Brinkac et al., 2019).

Clonal spread and horizontal transmission are the two most common carbapenemase-producing *Enterobacteriaceae* (CPE) dissemination methods. Compared with chromosomes, genes encoding carbapenemases are generally found in incompatible plasmid groups (Poirel et al., 2011). Horizontal transfer of resistance genes *via* mobile plasmids can accelerate the spread of resistance genes in diverse strains and hosts.

Plasmid incompatibility refers to the inability of two plasmids to coexist in the same cell, resulting in the loss of one plasmid in daughter cells, which is determined by replication machinery. In *Enterobacteriaceae*, 27 main plasmid incompatibility groups have been linked to antibiotic resistance genes (ARGs). A number of plasmid replicon groups in CREs have been reported globally, such as IncF, N, X, A/C, L/M, R, P, H, I, and W. Compared with other Inc. groups, IncF, A/C, and X are the most commonly associated with carbapenemase production.

IncX plasmids appear to be a potential reservoir for various resistance combinations, decreasing susceptibility to clinically important antimicrobials (Juraschek et al., 2021). IncX plasmids can be divided into nine subgroups based on repA sequences and binding sites (IncX1α, IncX1β, and IncX2-IncX8) (Fang et al., 2018). Among the IncX plasmid family, the IncX3 subgroup is primarily responsible for the dissemination of ARGs for clinically relevant first-line and last-resort (carbapenems) antibiotics (Liakopoulos et al., 2018). The prevalence of IncX3 plasmids was underestimated due to a lack of sequences available for setting PCR-based replicon typing (PBRT) until the *taxC* gene was used as a target to successfully identify the first IncX3 plasmid that represents the prototype IncX3 plasmid (Johnson et al., 2012). Then, until 2011, the first relevant properties of IncX3 plasmid pNDM-HN380 carrying *bla_NDM_* were recorded, which was one of the early steps in the evolution and spread of *bla_NDM_* (Ho et al., 2012). IncX3 plasmids have been implicated in the distribution of a range of *bla_NDM_* in humans, animals, and the environment in the last 10 years, particularly in Southeast Asia (Mouftah et al., 2019). IncX3 plasmid is an important vector carrying *bla_NDM_* (Wu et al., 2019; Touati et al., 2021). Furthermore, IncX3 is the most common subgroup found to harbor *bla_KPC_* and *bla_NDM_*, the two most prevalent carbapenemase genes (Kopotsa et al., 2019). The IncX3 plasmid has high stability, low fitness cost, great conjugation ability, and strong biofilm formation ability (Ma et al., 2020), all of which can facilitate the rapid and dominant dissemination of ARGs.

However, current research on IncX3 plasmids mainly focus on the epidemiological and molecular characteristics, while the physiological features of these plasmids are still unclear. Therefore, the purpose of this review is to (1) summarize the global epidemiological and molecular characteristics of IncX3 over the last 10 years, providing a theoretical basis for controlling the spread of IncX3 plasmids; and (2) organize the literature that have studied the physiological characteristics of IncX3 plasmids, providing directions and ideas for future IncX3 plasmid research.

## Literature search strategy

Publications chosen for this review were found on PubMed using the keywords “IncX3” as search criteria. As of May 21, 2022, a total of 273 articles were retrieved.

## Distribution of IncX3 plasmids in region, source, and host

**Figure 1:**
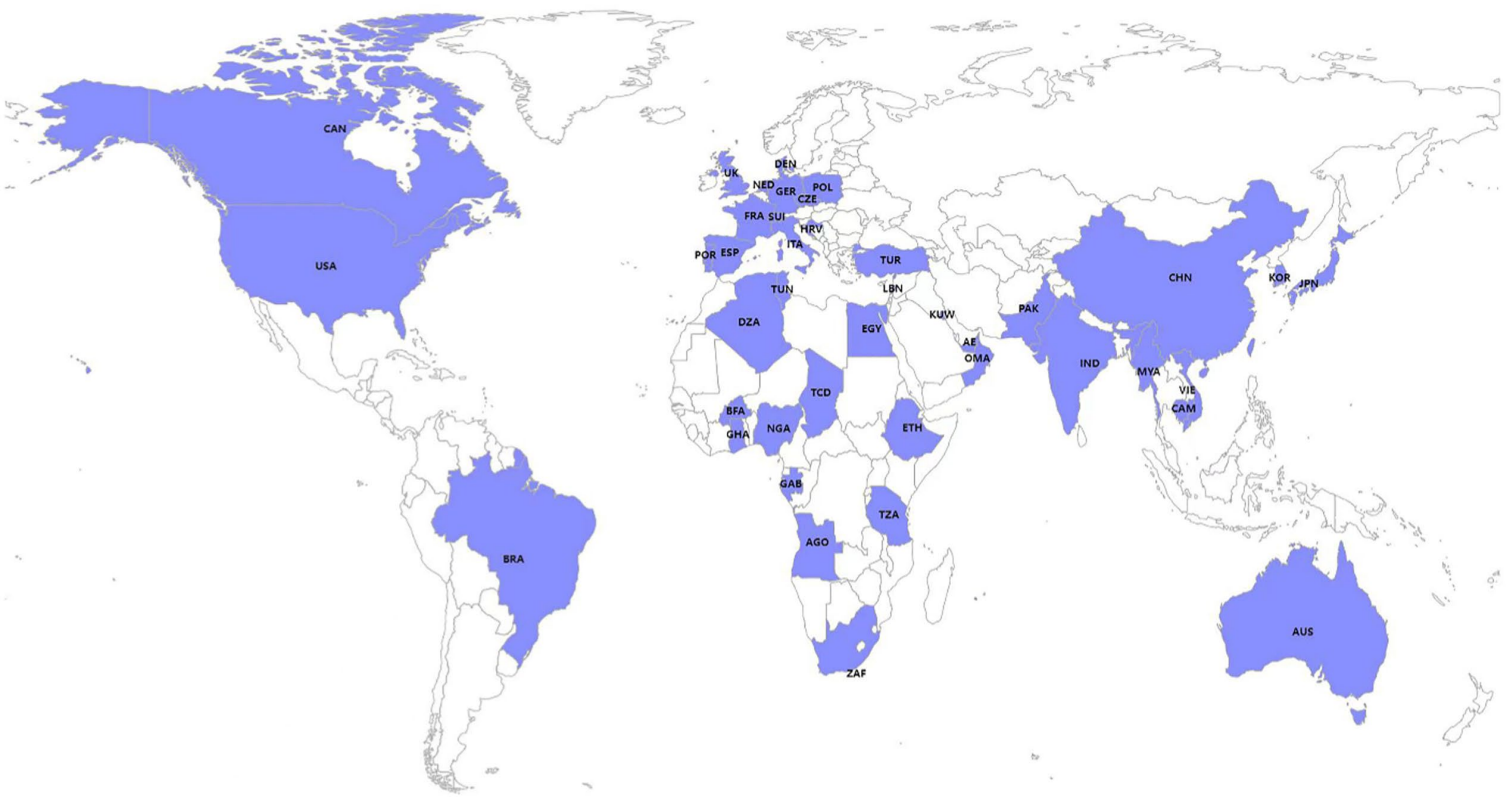
Global distribution of published strains harboring IncX3 plasmids included in this study.

Isolates harboring IncX3 plasmids are found in many countries around the world (Figure 1), including China (CHN), South Korea (KOR), Switzerland (SWI), Czech (CZE), Italy (ITA), Australia (AUS), India (IND), Japan (JPN), Netherlands (NED), Brazil (BRA), Germany (GER), South Africa (ZAF), Myanmar (MYA), the United States (USA), France (FRA), Ghana (GHA), Spain (ESP), the United Arab Emirates (AE), Cambodia (CAM), Canada (CAN), Denmark (DEN), Egypt (EGY), Kuwait (KUW), Pakistan (PAK), Poland (POL), Portugal (POR), the United Kingdom (UK), Oman (OMA), Vietnam (VIE), Algeria (DZA), Angola (AGO), Burkina Faso (BFA), Chad (TCD), Nigeria (NGA), Tanzania (TZA; Supplementary material), Tunisia (TUN; Dziri et al., 2022), Lebanon (LBN; Dagher et al., 2018), Ethiopia (ETH; Legese et al., 2022), Croatia (HRV; Petrosillo et al., 2016), Turkey (TUR; Peirano et al., 2022), and Gabon (GAB; Moussounda et al., 2017). Among them, China is the country with the most reported IncX3 plasmids.

The strains carrying IncX3 plasmids are from a rich source, mainly divided into clinical and non-clinical sources, with clinical sources accounting for two-thirds of the total (Supplementary material). Among them, urine ranked first, followed by blood, rectal swab, sputum, and stool specimens. In addition to the common specimens, clinical sources also include cerebrospinal fluid, bile, tracheal aspirate, drainage fluid, and wound. Non-clinical sources include livestock, environment (such as sewage treatment plants, rivers, and subway surfaces), companion animals, and food chains. Focusing on non-clinical specimens can help limit the threat of horizontal transfer to human health and take steps to regulate transmission routes and slow the global spread of antibiotic resistance genes.

One Health advocates that we should pay more attention to the interaction between humans, animals, and the shared environment, to achieve the best health outcomes, which is an important reason why we need to pay equal attention to the characteristics of IncX3 plasmids from non-clinical sources. Several investigations have shown interactions to mediate the spread of IncX3 plasmids in humans, animals, and the environment, resulting in the spread of resistance determinants. The *bla_NDM-5_*-producing *Escherichia coli* ST167 in both owners and companion animals suggests potential transmission between humans and dogs (Grönthal et al., 2018). IncX3 sequences in a *bla_NDM-5_*-positive strain isolated from a cat had areas with >99% nucleotide sequence identity to the human reference plasmid pNDM-MGR194 (Zhang et al., 2022). Furthermore, while carbapenems are not permitted for use in food-producing animals in China, the appearance of *bla_NDM-5_*-bearing IncX3 plasmids in swine-origin *K. pneumoniae* could be owing to environmental factors or human-to-livestock transmission (Zhao et al., 2021), but this hypothesis needs further confirmation. Meanwhile, the environment is also a potential reservoir for CRE as well as an essential source of transmission (Gu et al., 2022).

There are 23 species in the host of IncX3 plasmid (Supplementary material), including *Escherichia coli*, *Klebsiella pneumonia*, *Citrobacter freundii*, *Enterobacter cloacae*, *Citrobacter braakii*, *Citrobacter portucalensis*, *Citrobacter sedlakii*, *Cronobacter sakazakii*, *Enterobacter asburiae*, *Enterobacter hormaechei*, *Klebsiella quasipneumoniae*, *Klebsiella aerogenes*, *Klebsiella oxytoca*, *Klebsiella variicola*, *Kluyvera cryocrescens*, *Kluyvera intermedia*, *Morganella morganii*, *Proteus mirabilis*, *Raoultella ornithinolytica*, *Salmonella Typhimurium*, *Raoultella planticola*, and *Serratia marcescens*. Compared with other species, *E. coli*, *K. pneumonia*, *C. freundii*, and *E. cloacae* are the most commonly associated with IncX3 plasmid.

Among IncX3 plasmid-containing *Escherichia coli* and *Klebsiella pneumoniae* isolates, we discovered a surprising diversity of sequence types (STs). The most popular *E. coli* with distinct sequence types are ST410, ST167, ST48, and ST101. In Chinese clinical settings, ST167 *E. coli* strains had close ties to *bla_NDM-5_*, and high-level tigecycline resistance *Escherichia coli* carrying *bla_NDM-5_* also belonged to the ST167 clonal lineage (Li et al., 2018). This suggests that the ST167 is a major *bla_NDM-5_* reservoir in China. It is worth noting that ST48 only involves livestock sources, while ST101 only involves clinical sources. ST410 also includes clinical, animal, and environmental sources. The ST410 *Escherichia coli* carries diverse ARGs, indicating ST410 *Escherichia coli* is widely distributed and has a robust transmission and pathogenic potential. In *Klebsiella pneumoniae*, the most popular types are ST307, ST11, and ST15.

## Structural characteristics of IncX3 plasmids

IncX3 plasmids are narrow host range plasmids of *Enterobacteriaceae* (Zhu et al., 2020). The backbone and accessory module are the two main parts of the structure of the IncX3 plasmid. The core genes of the backbone include plasmid replication-related genes, conjugation-related genes, entry exclusion system-related genes, and partitioning system-related genes. The core genes are shared among the three typical completely sequenced IncX3 plasmids (pEC13_35, pIncX-SHV, and pNDM-HN380): replication (replication initiation protein, *pir*; replication accessory protein, *bis*), partitioning (*parA* and *parB*), plasmid maintenance (a putative DNA-binding protein, *hns*; a putative type III topoisomerase, *topB*), conjugation/type IV secretion system (T4SS, with 11 genes, *pilX1* to *pilX11*), transcriptional activator (*actX*), and putative DNA transfer proteins (*taxA* and *taxC*) (Ho et al., 2012). A retrospective analysis of IncX3 in China shows that the IncX3 plasmid backbone has been highly conserved over the past decade (Liu et al., 2021a). The accessory module mainly includes transposase genes, metabolic functions related genes, and antimicrobial resistance encoding genes. These are identified as acquired DNA sections coupled with and bordered by mobile elements and inserted at various backbone sites (Shi et al., 2018). In a study of IncX3 plasmids harboring *bla_NDM-5_* in retail beef in China (Zhang et al., 2019), a total of seven IncX3 genetic backgrounds were discovered. The variation is primarily due to mobile element insertions, truncation, and/or deletions. The conserved sequence of type I is IS3000-ΔISAba125-IS5-*bla_NDM_*-*ble_MBL_*-trpF-tat-Δdct-IS26-ΔumuD, which is the most common structure in *Enterobacteriaceae* of human, pig, and chicken sources in China and other countries. This shows that distinct mobile components are primarily responsible for the variety of the genomic environment in the IncX3 plasmid.

IncX3 plasmids have lower fitness costs. A study using the typical IncX3 plasmid pNDM-HN380 suggests that the IncX3 plasmid affected the motility of the host in addition to conferring multiple antibiotic resistance. Furthermore, the plasmid-encoded new sRNA IGR plas2 can influence fucose metabolism and biofilm formation to increase strain survival in a competitive environment (Huang et al., 2020). To our understanding, plasmids carrying resistance genes in strains impose fitness costs on their hosts in the absence of antibiotic selection pressure (Andersson and Hughes, 2010). However, in Ma’s study, 75.9% of wild-type strains receiving IncX3 plasmid demonstrated no fitness cost as a result of plasmid acquisition, and 6.9% of strains showed improved growth above their wild-type counterparts (Ma et al., 2020). The low fitness cost of the IncX3 plasmid may contribute to the stability of host bacteria and facilitate the horizontal transfer.

IncX3 plasmids are highly stable. In serial passage tests, the IncX3 plasmid is stable over time and confers a fitness benefit in selective antibiotic pressure (Flerlage et al., 2020). After 100 serial generations, IncX3 plasmids demonstrate remarkable stability in both clinical isolates and transconjugants, with no apparent plasmid loss (Tian et al., 2020). The IncX3 *bla_NDM-5_*-carrying plasmids in one *K. pneumoniae* clinical isolate showed high stability in clinical isolates, with no apparent plasmid loss after repeated cultures for 5 days (Zhu et al., 2020). Additionally, two *bla_NDM-5_* were found in *Escherichia coli* and were shown to be localized to the IncX3 and IncFII plasmids, respectively, in a study. Transconjugants carrying the IncX3 plasmid had higher levels of carbapenem resistance and *bla_NDM-5_* expression than those carrying both plasmids or only carrying the IncFII plasmid (Yang et al., 2020).

IncX3 plasmids are conjugative and could enhance biofilm formation. Research suggests that IncX3 plasmid can transfer *bla_NDM_* between different enterobacterial species over a wide temperature range (Liu et al., 2019). And this is attributed to the type IV secretion system encoded by the IncX3 plasmid, which, in addition to providing transfer functions for the plasmid, can also provide auxiliary functions such as resistance gene acquisition and biofilm formation to its host strains (Johnson et al., 2012; Wang et al., 2018). Biofilms can improve bacteria’s antibiotic resistance and raise their tolerance to harsh environmental conditions (Rabin et al., 2015). The acquired resistance to β-lactam antibiotics and heightened biofilm-forming potential of the wild-type strain after receiving the IncX3 plasmid may impair the drug’s efficacy (Ma et al., 2020). Transferring this plasmid to extremely virulent bacteria may improve pathogenicity and multidrug resistance, potentially increasing treatment failure rates (Ma et al., 2020). However, some studies have demonstrated that IncX3 plasmids do not promote bacterial pathogenicity (Liakopoulos et al., 2018; Liu et al., 2020). This could be related to the virulence and pathogenicity of the recipient strain itself, which requires further research.

The conjugation capacity of the IncX3 plasmid is inextricably linked to its high stability, and inhibiting conjugation transfer can lead to the elimination of the plasmid from the strain (Zhu et al., 2020). Plasmid stability can be improved further *via* IncX3 plasmid conjugation transfer. Linoleic acid is a conjugative transfer inhibitor. It can limit the frequency of plasmid conjugation and induce the loss of IncX3 plasmids in strains (Zhu et al., 2020). This conclusion reveals that conjugative transfer inhibitors can lessen the two key benefits of IncX3 plasmids, which is an excellent strategy to keep plasmids from spreading.

The majority of IncX3 plasmids are between 30 kb and 60 kb in size, with a few between 60 kb and 70 kb. The smallest plasmid in this collection, pKB11 (accession number: MK264769), is 12,757 kb in size (Fuga et al., 2020). Only sequences encoding replication, resistance (*bla_KPC-2_* on NTEKPC-Ic), and auxiliary genes were found in the basic structure of pKB11. Although this novel tiny plasmid lacks conjugation genes, the host of pKB11 contained additional plasmids from various incompatibility groups, which may aid the transfer of this novel IncX3 plasmid to a new host. Following that was an unnamed3 plasmid (accession number: MN847777) with 18,178 bp but no conjugation genes (Cao et al., 2020). This is nearly identical to a non-conjugative 17-kb IncX3 plasmid harboring *bla_NDM_*. It eliminated large-scale regions compared to the typical IncX3 plasmid, suggesting a low fitness cost dissemination strategy for the IncX3 plasmid (Li et al., 2022). The 75,415-bp plasmid pWLK-NDM (accession number: CP038280) is the largest in this collection. In China, this plasmid was isolated from urban river debris. Despite having a conjugation gene, it is unable to conjugate due to the disruption of the *pilX3-4* gene. This plasmid is also significantly larger than other IncX3 plasmids, which could be the result of genetic exchange between different bacterial species from various environments (Dang et al., 2020).

## Various antibiotic resistance genes carried by IncX3 plasmids

**Figure 2:**
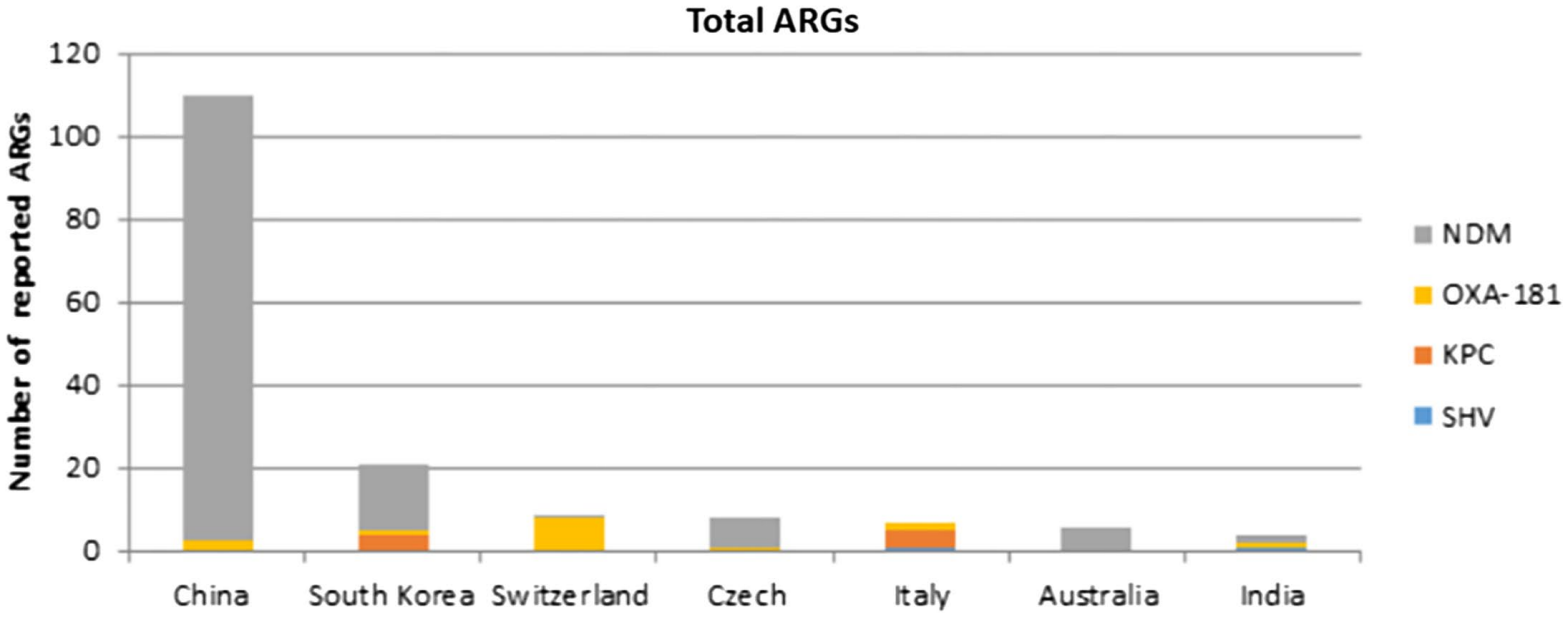
Number of carbapenemase genes reported in different countries represented by this review.

**Figure 3:**
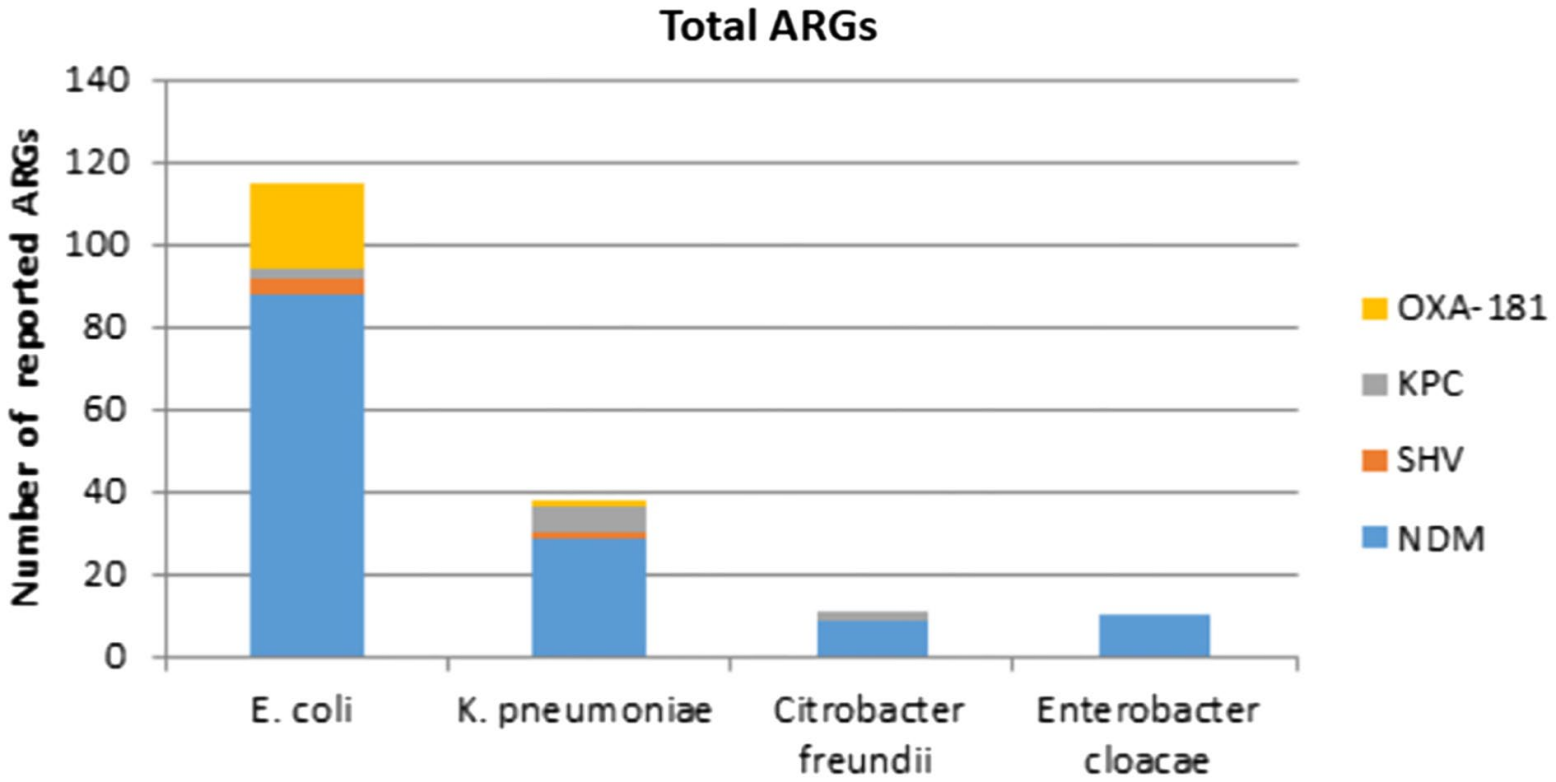
Number of carbapenemase genes reported in different species represented by this review.

All IncX3 plasmids cited in this review carry ARGs, the majority of which are connected to carbapenemase genes. *bla_NDM_* had the highest prevalence, followed by *bla_OXA-181_* and its variants, *bla_KPC_*, and *bla_SHV_*. Interestingly, the majority of gene types carried on plasmids in China are *bla_NDM_* and its variations. Except for *bla_NDM-4_*, all *bla_NDM_* variants located on IncX3 plasmids can be found in China. And no IncX3 plasmid carrying *bla_KPC_* was recorded in China. The frequency distribution of carbapenemase genes reported in different nations and species covered by this review are shown in Figures 2, 3.

The IncX3 plasmids carry various NDM variants, including NDM-5, NDM-1, NDM-7, NDM-4, NDM-11, NDM-13, NDM-17, NDM-19, NDM-20, NDM-21, and NDM-33 (Supplementary material). This suggests that the *bla_NDM_*-bearing IncX3 plasmids descended from a common ancestor plasmid through a series of mutations. The easy propagation of the IncX3 plasmid could be responsible for the spread of various NDM variants in *Enterobacteriaceae* isolates (Tian et al., 2020). NDM-positive strains can carry multiple types of plasmids, with IncX3 plasmids accounting for roughly one-third of the plasmids in the gene bank (Ma et al., 2020). The IncX3 plasmid is not only the most common vehicle for harboring *bla_NDM_* but may also be one of the most important platforms for *bla_NDM_* evolution as novel NDM variants emerge (Wu et al., 2019). Notably, *bla_NDM-5_*-carrying IncX3 plasmid was the most common type in *bla_NDM_*, followed by *bla_NDM-1_*-carrying IncX3 plasmid, which is consistent with the findings of Kopotsa et al. (2019). NDM-1 and NDM-5 were found to be more frequently connected with IncX3 plasmids than other NDM variations, according to the researchers. NDM-1 and NDM-5 differ in amino acid sequence at positions 88 (ValLeu) and 154 (MetLeu) and NDM-5 has a greater carbapenem hydrolysis ability (Sun et al., 2019).

Notably, we found conserved sequences in the genetic environment of most *bla_NDM_* and its variants. The majority of the strains have the conserved structure “*bla_NDM_*-*ble_MBL_*-trpF-dsbC,” but some simply have the “*bla_NDM_*-*ble_MBL_*-trpF” structure, or the dsbC was changed into the dcbD “*bla_NDM_*-*ble_MBL_* -trpF-dcbD,” and only two strains carrying *bla_NDM-5_* had the conserved sequence” *bla_NDM_*-*ble_MBL_* -trpT-dcbD.” This conserved sequence may play a key role in stability and retention, increasing the transfer of ARGs and improving enzyme activity. However, the specific functions of related genes need to be investigated further (Brinkac et al., 2019).In addition, the environment surrounding *bla_NDM_* contains not only conserved sequences but also mobile elements such as ISAba125, IS3000, IS5, and IS26, indicating that *bla_NDM_* acts as an external gene to recombine into the IncX3 plasmid by insertion or transposition depending on IS elements (Liu et al., 2021b). Meanwhile, IS26 is linked to *bla_SHV_*, *bla_KPC_* to ISKpn6 and ISKpn7, *bla_NDM_* to ISAba125 (Sun et al., 2019), *bla_OXA-181_* to IS26 and ISEcp1 (Supplementary material). This is consistent with Li et al. (2020). They claim that the IncX3 plasmid contains a changeable area that primarily encodes resistance to clinically significant antimicrobial agents and encodes genes required for its maintenance and dissemination. The findings confirm the potential of IncX3 plasmid for accumulating resistance genes *via* IS-mediated transposition, with the likely consequence of limiting effective treatment options for possible human infections. In *bla_NDM_*-carrying IncX3 plasmids, deletion events with a 500-bp deletion in ISAba125 and a 180-bp deletion in dsbC were observed, which is significant and powerful evidence that ISAba125 and dsbC were unstable, implying that *bla_NDM_* is evolving to reduce the burden to facilitate rapid and stable horizontal transfer (Liu et al., 2021b).

We also observed IncX3 plasmids to examine if they also carried other ARGs. Besides harboring *bla_NDM_*, *bla_KPC_*, and *bla_SHV_*, most IncX3 plasmids carry no additional antibiotic resistance genes, especially IncX3 *bla_NDM-5_*-carrying plasmids (García-Fernández et al., 2012; Chakraborty et al., 2021; Lu et al., 2022). The size of the plasmid likely limits the number of antibiotic resistance genes that the plasmid can carry. IncX3 plasmid carrying only *bla_NDM_* is similar to the original plasmid pNDM-MGR194 (accession number: KF220657) (Krishnaraju et al., 2015), which is an early prototype in the dissemination of *bla_NDM_*. This is the first report of *bla_NDM_* being inserted into a plasmid without the presence of other resistance gene determinants. Interestingly, all plasmids carrying *bla_OXA-181_* also carry the qnrS1, while plasmids carrying *bla_NDM-1_* often also carry *bla_SHV-12_*. This is consistent with the study by Xiang et al. (2021). IncX3 plasmids can disseminate *bla_OXA-181_* and qnrS *via* an IS26-flanked composite transposon, or *bla_NDM_* and *bla_SHV-12_ via* a Tn125-like transposon. And these are the two main epidemic types of IncX3 plasmids.

## Functional study of related genes in IncX3 plasmid

The IncX3 plasmids with conjugation ability accounted for the majority (Kim et al., 2020; Ma et al., 2020; Zhu et al., 2020). The deletion or destruction of conjugation genes may be the reason for IncX3 plasmids losing conjugation ability (Pál et al., 2017; Di Pilato et al., 2020). However, some plasmids still failed to transfer in conjugation studies despite having entire backbone structures (Wang et al., 2017; Dang et al., 2020). The cause for this remains unknown. The IncX3 plasmids have a conjugation frequency of between 10^−9^ and 10^−2^ (Liu et al., 2017; Lu et al., 2022). The short size of the IncX3 plasmid may also play a part in the high transfer frequency, and such a high frequency and stable conjugation ability have played a key role in the rapid and widespread dissemination of IncX3 over the last decade.

The conjugation frequency of IncX3 plasmid varies in different strains, according to current research on the properties of IncX3 plasmid and gene function. Varying ST strains have different conjugation frequencies, even within the same strain. The IncX3 plasmid in the donor strain is inaccessible to *Acinetobacter baumannii* and *Pseudomonas aeruginosa* as receivers for conjugative transfer (Wang et al., 2018). The frequency of conjugation varies among strains from different sources. The conjugation frequency of animal-derived plasmids is lower at 37°, whereas the conjugation frequency of human-derived plasmids is higher (Liakopoulos et al., 2018). In Baomo’s study, four different temperatures were used to investigate the role of IncX3 plasmids in frequency, fitness cost, and stability at different temperatures: environment temperature in Asia and the Middle East (25 and 30°), human body temperature (37°), and chicken body temperature (42°; Sugawara et al., 2019). The data demonstrate that at 37°, the IncX3 plasmid has a higher transfer frequency, more stability, and a lower fitness cost, indicating that the plasmid has the highest transfer in human and other mammalian intestines and has been adapted to exist in the mammalian gut. This could explain why the IncX3 plasmid can be found in humans and other mammals, as well as in bacteria with different antibiotic resistance genes. The IncX3 plasmids had conjugation frequencies equivalent to or higher than the reference IncFII plasmid at 30 and 37° (Wang et al., 2018). IncX4 conjugal transfer to recipient *E. coli* at 30° is also higher than IncFII, demonstrating that this trait is common to all IncX plasmid subtypes (Wang et al., 2018).

Ho et al. hypothesized that the IncX3 plasmid backbone contains a histone-like nucleoid structuring (H-NS)–like protein that stabilizes plasmid DNA and regulates plasmid transfer at different temperatures (Ho et al., 2012). Furthermore, Sugawara et al. recently discovered that *hns* genes are ubiquitous in IncX3 plasmids, implying that H-NS-like proteins may influence the propagation of IncX3 plasmids (Sugawara et al., 2019). The role of the *hns* gene in plasmid dispersion, stability, fitness cost, and temperature regulation was fully clarified by Baomo’s study (Liu et al., 2020; Baomo et al., 2021). The author claims that the IncX3 plasmid, which lacks the plasmid-encoded *hns* gene, can increase conjugation frequency by upregulating conjugation-related genes and that the *hns* gene regulates conjugation transfer temperature-dependently. By boosting the expression of the *parB* gene, H-NS-like proteins can also improve plasmid stability in the host. H-NS-like protein also impacts the virulence of the plasmid by regulating the virulence gene encoded by the chromosome to create fimbriae for the adhesion and invasion of the strain.

## Cointegrate plasmids associated with IncX3 plasmids

Strains carrying IncX3 plasmids tend to carry more than one plasmid. Inc. typing is a method of classifying plasmids based on their ability to coexist stably with other plasmids in the same bacterial strain, determined by their replication machinery. When co-resident plasmids have the same replication mechanisms, they are incompatible (Johnson and Nolan, 2009). The number of plasmids carried by the strains ranged from 1 to 8 plasmids (Supplementary material), with 2, 3, and 4 being the most common. Multiple plasmids in the same strain are uncommon (e.g., 7–8), and the coexistence of a large number of plasmids reflects that active horizontal transfer events may have occurred before.

In most cases, a plasmid will only contain one type of replicon gene. Recent research has found that large MDR conjugative plasmids can harbor numerous replicon genes, expanding the host range of such plasmids. Furthermore, mobile elements are the main contributors to plasmid replicon fusion. Plasmid evolution by combining two or more MDR plasmids would increase the resistance profile of the resulting plasmid. Moreover, it would broaden the host spectrum of such resistance-encoding mobile elements, limiting antibiotic selection and application in clinical practice. Increasing the fitness cost could, in general, limit the spread of cointegrate plasmids. In high-risk strains, however, cointegrate plasmids remained stable in the presence of antibiotics, implying that drug residue is an essential driving force in the evolution of MDR pathogens (Liu et al., 2021c). Cointegrate plasmids have more ARGs and insertion sequences with high plasticity than regular plasmids because of the integration of genetic information from original plasmids. In response to a complicated environment, the cointegrate plasmid could dynamically discard some areas and acquire new ARGs, exhibiting its great dispersion and evolution potential (Liu et al., 2021c). The explosive proliferation of cointegrate plasmids in *Klebsiella pneumoniae* was observed by the HIN Hospital in the United States, which could be a means for plasmids to seek stable forms in evolution (Liu et al., 2021c). Transferring this plasmid to clinic-associated strains could improve biofilm formation capabilities, resulting in higher treatment failure rates (Hargreaves et al., 2015). Cointegrate plasmids should therefore be given special attention. A better understanding of the molecular mechanisms underlying the formation and evolution of cointegrate MDR plasmids will pave the way for preventing the spread of resistance elements among bacterial pathogens *via* IncX3 plasmid.

The IncX3 plasmid, as a typical self-transfer plasmid, can behave as a helper plasmid, assisting other non-conjugative plasmids in their transfer to other bacterial hosts and spreading antibiotic resistance genes further (Conlan et al., 2014). IncA/C, FII, FIA, FIB, IncR, IncY, and ColE plasmids are among the plasmids that cointegrate with IncX3 plasmids. The IS26 element plays a significant role in creating cointegrate plasmids, and its insertion promotes the dissemination of antibiotic resistance genes and the formation of cointegrate plasmids (Hao et al., 2021). Plasmid stability experiments on *Salmonella* containing IncX3 and IncA/C cointegrate plasmids revealed that cointegrate plasmids had high stability but were easily decomposed into single plasmids during conjugation and transfer, and the decomposed IncX3 plasmid could still transfer to the recipient strain (Li et al., 2020). *K. aerogenes* was found to have the IncC-IncX3 cointegrate plasmid pNUITM-VK5_mdr (accession number: LC633285), which was resistant to practically all antibiotics, including tigecycline, tetracyclines, carbapenems, cephalosporins, fluoroquinolones, and aminoglycosides (except amikacin) (Hirabayashi et al., 2021). The fusion is thought to have an increased risk of carrying more ARGs and spreading more stably and efficiently among bacteria in humans, animals, and the environment. Hence, IncX3 plasmids with high stability, low fitness cost, and self-transferability can assist broad-host-range plasmids that carry multiple antibiotic resistance genes but do not have the conjugation ability to achieve their transfer and dissemination. In return, broad-host-range plasmids may also promote the stabilization of co-resident IncX3 plasmid (Bitar et al., 2019).

## Conclusion

Carbapenem resistance is a clinical problem to be solved urgently. The ability of antibiotic-resistant strains to produce carbapenemase is an important mechanism of carbapenem resistance, while plasmids play an important role in spreading and carrying antibiotic resistance genes. Among these, the IncX3 plasmid has low fitness costs, high stability, high transfer frequency, and improved strain biofilm-forming capabilities. The IncX3 plasmid is crucial for transporting and spreading carbapenemase genes (*bla_NDM_*, *bla_KPC_*, and *bla_OXA_*), particularly *bla_NDM_*. IncX3 plasmid is not only a vehicle for spreading *bla_NDM_* but also a platform for evolution. Many NDM variations, particularly *bla_NDM-5_*, can be found in IncX3. IncX3 plasmids have been isolated from different human, animal, and environmental strains worldwide, especially in China. The plasmids are primarily found in *E. coli*, *K. pneumoniae*, *C. freundii*, and *Enterobacter cloacae.* There are usually 1–3 co-resident plasmids in the IncX3 plasmid, with the transfer frequency ranging from 10^−9^ to 10^−2^ and the size of the plasmid usually ranging from 30 to 60 kb. It can be used as a co-resident plasmid to form a fusion with a non-self-transfer plasmid, and IncX3 plasmids could help expand the resistance profile and disseminate resistance genes. The structure of the IncX3 plasmid is divided into two parts: the backbone and the accessory module. The backbone is highly conserved, and its related genes play an important role in the stability and spread of the plasmid. More research into the physiological aspects is still needed. Based on this, future research should focus on the physiological role of the plasmid structure to provide a theoretical basis for controlling the global prevalence of IncX3.

## Author contributions

XG contributed to the conceptualization. RC, CL, and HG searched articles and collected data. JQ and YL analyzed the data. RC and QW contributed to the writing and final approval. All authors contributed to the article and approved the submitted version.

## Funding

This work was supported by grants from the Henan Province Medical Science and Technology Research Project Joint Construction Project (no. LHGJ20190232) and the Youth Innovation Fund project of the First Affiliated Hospital of Zhengzhou University (no. YNQN2017168).

## Conflict of interest

The authors declare that the research was conducted in the absence of any commercial or financial relationships that could be construed as a potential conflict of interest.

## Publisher’s note

All claims expressed in this article are solely those of the authors and do not necessarily represent those of their affiliated organizations, or those of the publisher, the editors and the reviewers. Any product that may be evaluated in this article, or claim that may be made by its manufacturer, is not guaranteed or endorsed by the publisher.
